# A Computed Tomography-Based Radiomics Nomogram to Preoperatively Predict Tumor Necrosis in Patients With Clear Cell Renal Cell Carcinoma

**DOI:** 10.3389/fonc.2020.00592

**Published:** 2020-05-29

**Authors:** Yi Jiang, Wuchao Li, Chencui Huang, Chong Tian, Qi Chen, Xianchun Zeng, Yin Cao, Yi Chen, Yintong Yang, Heng Liu, Yonghua Bo, Chenggong Luo, Yiming Li, Tijiang Zhang, Rongping Wang

**Affiliations:** ^1^Medical College, Guizhou University, Guiyang, China; ^2^Department of Medical Records and Statistics, Guizhou Provincial People's Hospital, Guiyang, China; ^3^Department of Radiology, Guizhou Provincial People's Hospital, Guiyang, China; ^4^Guizhou Provincial Key Laboratory of Intelligent Medical Image Analysis and Precision Diagnosis, Guizhou Provincial People's Hospital, Guiyang, China; ^5^Research Collaboration Department, R&D Center, Beijing Deepwise & League of PHD Technology Co.LTD, Beijing, China; ^6^Department of Pathology, Guizhou Provincial People's Hospital, Guiyang, China; ^7^Department of Radiology, Affiliated hospital of Zunyi Medical University, Zunyi, China; ^8^Department of Pathology, Affiliated hospital of Zunyi Medical University, Zunyi, China; ^9^Department of Urinary Surgery, Guizhou Provincial People's Hospital, Guiyang, China

**Keywords:** clear cell renal cell carcinoma, tumor necrosis, computed tomography, radiomics, prediction model

## Abstract

**Objective:** To develop and validate a radiomics nomogram for preoperative prediction of tumor necrosis in patients with clear cell renal cell carcinoma (ccRCC).

**Methods:** In total, 132 patients with pathologically confirmed ccRCC in one hospital were enrolled as a training cohort, while 123 ccRCC patients from second hospital served as the independent validation cohort. Radiomic features were extracted from corticomedullary and nephrographic phase contrast-enhanced computed tomography (CT) images. A radiomics signature based on optimal features selected by consistency analysis and the least absolute shrinkage and selection operator was developed. An image features model was constructed based on independent image features according to visual assessment. By integrating the radiomics signature and independent image features, a radiomics nomograph was constructed. The predictive performance of the above models was evaluated using receiver operating characteristic (ROC) curve analysis. Furthermore, the nomogram was assessed using calibration curve and decision curve analysis.

**Results:** Thirty-seven features were used to establish a radiomics signature, which demonstrated better predictive performance than did the image features model constructed using tumor size and intratumoral vessels in the training and validation cohorts (p <0.05). The radiomics nomogram demonstrated satisfactory discrimination in the training (area under the ROC curve [AUC] 0.93 [95% CI 0.87–0.96]) and validation (AUC 0.87 [95% CI 0.79–0.93]) cohorts and good calibration (Hosmer-Lemeshow p>0.05). Decision curve analysis verified that the radiomics nomogram had the best clinical utility compared with the other models.

**Conclusion:** The radiomics nomogram developed in the present study is a promising tool to predict tumor necrosis and facilitate preoperative clinical decision-making for patients with ccRCC.

## Introduction

Renal cell carcinoma (RCC) is the most common malignant neoplasm of the kidney in adults, of which clear cell RCC (ccRCC) is the most prevalent subtype, accounting for 70–80% of neoplasms (1, 2). Tumor necrosis is defined as coagulation necrosis of tumor cells observed by microscopy, exhibiting characteristics of dead and degraded tumor cells formed into homogeneous clusters and sheets (3, 4). However, histopathological features, including hemorrhage, cystic transformation, hyalinization, as well as foci of fibrosis, should not necessarily be regarded as tumor necrosis (3). Numerous studies have demonstrated that the presence of tumor necrosis is a reflection of aggressive behavior and an independent predictor of poor survival in patients with ccRCC (5, 6). Therefore, The International Society of Urological Pathology (ISUP) recommended that tumor necrotic pathological information should be routinely included in pathological reports for ccRCC (7). Furthermore, tumor necrosis can enhance the prognostic performance of other prognostic variables including tumor size, TNM stage, and nuclear grade in prognostic algorithms, the most well-known of which is the Mayo Clinic Stage, Size, Grade and Necrosis (SSIGN) score (8, 9). It is becoming increasingly important to obtain accurate prognostic information and to accurately assess tumor aggressiveness before treatment to determine the optimal treatment strategy (4, 10). However, information regarding tumor necrosis is available only after surgical pathological evaluations. Although preoperative biopsy provides important histological information, it has some limitations, including insufficient accuracy, resulting in sampling bias and the risk for significant complications (11). Therefore, a non-invasive and accurate method to predict tumor necrosis in patients with ccRCC before treatment is urgently needed.

Computed tomography (CT) is generally considered a common non-invasive imaging modality for preoperative tumor staging and assessing aggressiveness in patients with ccRCC (12). However, the accuracy of visually assessing CT images is extremely limited by the subjectivity and experience of the radiologists (13). Recent studies have proposed that images are more than pictures; they are, in fact, data (14). An emerging field, known as radiomics, proposes a new concept for precision medicine based on medical images, the methodology of which is to extract large numbers of quantitative features from images to describe tumor phenotypes using advanced mathematical algorithms (15, 16). ccRCC is a highly heterogeneous tumor, with which radiomic features demonstrate an excellent correlation (17, 18). Subsequently, recent advances have shown that radiomics holds great promise in evaluating and predicting histopathological features and treatment response (19–21). To date, however, the feasibility of CT-based radiomics models in preoperatively predicting tumor necrosis in patients with ccRCC has not been evaluated.

Therefore, the purpose of this study was to evaluate the performance of radiomics signature and image features model in preoperatively predicting tumor necrosis, and to establish a radiomics nomogram integrating radiomics signature and independent image features, which is expected to categorize tumor necrosis accurately and effectively guide individualized treatment in patients with ccRCC.

## Materials and Methods

### Participant Selection

All patients were consecutively enrolled between August 2013 and December 2017 at Guizhou Province People's Hospital (GzPPH; Guiyang, China) or between February 2010 and December 2017 at the Affiliated Hospital of Zunyi medical University (AHZMU; Zunyi, China). Inclusion criteria were as follows: postoperative pathological diagnosis of ccRCC; not having undergone any treatment before operation; and availability of complete contrast-enhanced CT imaging data within 30 days before the operation. Individuals in whom percutaneous renal mass biopsy was performed before CT enhancement examination, those with Ct images with obvious noise and artifacts, and those with incomplete imaging, clinical or pathological data were excluded.

Demographic data, including age and sex, and pathological information were retrieved from the electronic medical records system. The corticomedullary, nephrographic phase contrast-enhanced CT images from all patients were retrieved and downloaded from the image archiving and communication system and saved in dicom format for further analysis The ct scans from the two centers involved in this study were performed using two different CT scanners. Specific details of the CT equipment and parameters are detailed in Supplementary Materials.

### Pathological Assessment

Tumor necrosis in ccRCC from different hospitals was reviewed by two senior pathologists, Y.Y.T (from GZPPH, with 21 years' experience in pathological diagnosis) and B.Y.H. (from AHZMU, with 14 years' experience in pathological diagnosis), according to the 2016 World Health Organization (WHO) system based on the consensus conference of the ISUP. Although the two physicians knew that all cases were ccRCC, they were blinded to the diagnosis of tumor necrosis.

### Subjective Image Features Analysis

CT images from all cases were reviewed by two attending radiologists (Z.X.C. and L.H., GPPH and AHZMU, with 19 years and 11 years' experience in abdominal imaging diagnosis, respectively). They assessed the imaging features of the cases in their respective hospitals. All physicians knew that the tumor was diagnosed as ccRCC, but were blinded to the pathological diagnosis of tumor necrosis.

**Figure 1:**
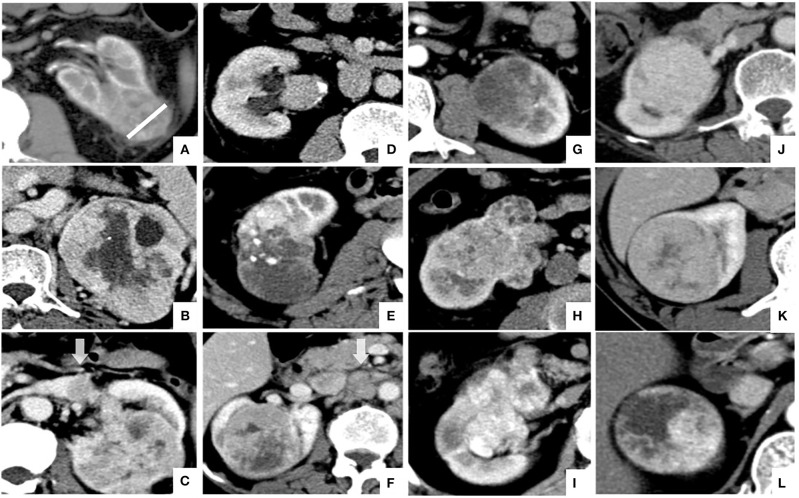
Illustration of CT features of CCRCC in axial images: **(A)** tumor size (white line) and blurred tumor boundary; **(B)** necrosis imaging; **(C)** renal vein invasion; **(D)** collecting system invasion; **(E)** intratumoral vessels; **(F)** positive lymph node metastasis; **(G–I)**; visual relative enhancement: **(G)** hypoattenuating, **(H)** isoattenuating, **(I)** hyperattenuating; **(J–L)** enhancement pattern: **(J)** homogeneous enhancement, **(K)** relative homogeneous enhancement, **(L)** heterogeneity enhancement.

The image features assessed in the present study were as follows: tumor size, defined as the maximum diameter of the tumor at the axial level; tumor boundary, divided into clear boundary and blurred boundary according to the signs of infiltration around the tumor in the nephrographic phase; necrosis imaging, defined as the non-enhanced liquid area of the tumor is >50% of the tumor; renal vein invasion, defined as the imaging feature of tumor thrombus in renal vein and inferior vena cava; collecting system invasion, defined as deformation of the collection system or tumor invading the renal pelvis and renal cone; intratumoral vessels, defined as visible vascular enhancement in the corticomedullary phase; positive lymph node metastasis, defined as the short-axis diameter of lymph nodes >10 mm in the renal hilum and retroperitoneum; visual relative enhancement, divided into hyperattenuating (more obvious than renal cortex enhancement), isoattenuating (similar to renal cortical enhancement), and hypoattenuating (weaker than renal cortical enhancement) (7); and enhancement pattern, divided into homogeneous enhancement (90%), relative homogeneous enhancement (75–90%), and heterogeneity enhancement (<75%) according to the homogeneous of tumor enhancement (7). A representative example of the above imaging features is shown in Figure 1.

### Image Features Model Building

Candidate indicators of image features models included the following: age, sex, tumor size, imaging necrosis, renal vein invasion, collective system invasion, intra-tumoral vessels, positive lymph node metastasis, enhancement mode, and relative visual enhancement. Univariate analysis was used to assess the correlation between the above indicators and tumor necrosis in the training cohort. Important risk indicators in the univariate analysis (i.e., those with *p* < 0.05) were included in the multivariate logistic regression analysis to identify independent risk indicators. A predictive model of image features was then constructed in the training cohort and confirmed in the verification cohort.

### Tumor Segmentation

This study used ITK-SNAP version 3.8 software (www.itksnap.org) to perform three-dimensional manual segmentation of the tumor region of interest (ROI). First, an attending radiologist (T.C., GZPPH, 6 years' experience in abdominal diagnosis) identified tumor boundaries based on CT multi-phase enhancement images. Then, the ROI was outlined along the borders of the tumors on the corticomedullary and nephrographic phases, while avoiding covering adjacent vessels, perirenal fat, and the renal parenchyma. Finally, the senior radiologist (Z.X.C) reviewed all ROI segmentation.

### Radiomics Feature Extraction

This study used the Dr. Wise Multimodal Research Platform (https://research.deepwise.com) (Beijing Deepwise & League of PHD Technology Co., Ltd, Beijing, China) for feature extraction in the training cohort. First, pre-processing was performed using B-spline interpolation resampling techniques, resampling all of the CT images such that they were 0.75 × 0.75 × 0.75 mm^3^ voxels. Then, 1,218 features were extracted from the ROI on corticomedullary, nephrographic phase contrast-enhanced CT images. The extracted radiomics features were divided into three categories: features based on tumor shape and size; first-order gray-scale statistical features; and texture-based features, including gray-scale co-occurrence matrix (GLCM), gray-level size zone matrix (GLSZM), gray level run length matrix (GLRLM) and gray level difference matrix (GLDM). Moreover, the whole radiomic feature set also contained higher order statistical features, including the intensity and texture features derived from the images processed with 2 types of filters (logarithm and wavelet transformation).

### Radiomics Signature Construction

The corticomedullary and nephrographic features were combined and analyzed because different contrast-enhanced phases can characterize tumor different biological information. High-dimensional data may contain highly redundant and uncorrelated information, which may lead to over-fitting and reduce the performance of the learning algorithm. Feature dimension reduction and screening were then performed in two steps.

In the first step, intra- and interobserver intraclass correlation coefficient (ICC) were used to screen radiomics features with better robustness in feature extraction. Thirty randomly selected patients were used to test the ICC, 15 patients of them from the training cohort and 15 patients from the validation cohort. The radiological attending physician (T.C.) and the senior physician (Z.X.C.) independently delineated the ROI for the corticomedullary and nephrographic phases of the 30 patients. Two weeks later, the radiologist repeated the two ROIs. The consistency of the extracted features was based on ROI delineation between the intra-observer and interobserver. Features with ICC > 0.75 were considered to be consistent and retained for further analysis.

In the second step, the radiomic features were standardized by the standard scaler package in tranning cohort. The mean of features set was mapped to zero, and the standard deviation mapped to one in the process of features standardization. Then, the standardized model in the training cohort was applied to the validation cohort. Then using least absolute shrinkage and selection operator (LASSO) logistic regression, the best feature data set with the smallest binomial deviation was selected by 10 fold cross-validation, and the radiomics features with significant coefficient non-zero and tumor necrosis was screened out. Based on the LASSO weighting coefficients of the selected features, radiomics features were linearly combined to construct a radiomics score (Rad score) formula (i.e., radiomics signature). Based on this formula, a risk score can be calculated for each patient that reflects the predicted risk of imaging histology labeling for the presence of tumor necrosis. In the training set, the best cut-off value for the Rad score was statistically analyzed using the Youden index, then the patients were divided into high-risk groups (with tumor necrosis) or low-risk groups (non-tumor necrosis). Finally, the verification of radiomics signature was performed in the verification set.

### Radiomics Nomogram Construction

To provide patients and clinicians with an individualized and easy-to-use preoperative predictive tool for tumor necrosis, this study constructed a radiomics nomogram. The radiomics nomogram integrated the radiomics signature and independent image features. Then, the multicollinearity analysis between variables in the model based on variance inflation factor (VIF). Finally, the nomogram was tested in the verification cohort.

### Model Evaluation

**Figure 2:**
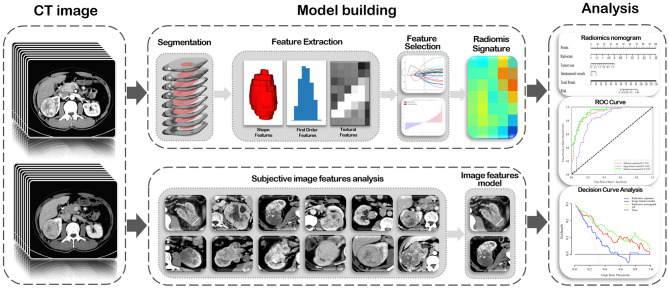
A flowchart of radiomics analysis in this study.

ROC curves were plotted and area under the ROC curve (AUC) was used to evaluate the predictive performance of the radiomics signature, image feature model, and radiomics nomogram for tumor necrosis in ccRCC in the training and validation cohorts. The optimal cut-off values for the different models were evaluated using the Youden index, and differences in AUC values among the three models were compared using the Delong test in both cohorts. Consistency of the predicted risk for tumor necrosis using actual results of the radiomics nomogram was demonstrated by a calibration curve. And the calibration of the nomogram was evaluated through the Hosmer-Lemeshow goodness-of-fit test with 8 groups. To assess the clinical usefulness of the radiomics nomogram, decision curve analysis was used to quantify the net benefit at different threshold probabilities in the validation cohort. A flowchart of radiomics analysis is shown in the Figure 2.

### Statistical Analysis

Statistical analysis was performed using SPSS version 21.0 (IBM Corporation, Armonk, NY, USA), R (version 3.4.0; http://www.r-project.org), or Python version 3.6.8 (https://www.python.org). Continuous variables are expressed as mean ± standard deviation and categorical variables are expressed as number (percent [%]). In the univariate analysis, the continuous variables were tested using *t*-test or Mann–Whitney *U*-test, while categorical variables were analyzed using the chi-squared test or Fisher's exact test; a two-sided *p* < 0.05 was considered to be statistically significant. Intra- and interobserver consistency of features extracted from the ROIs was assessed using Kappa statistics. The scikit-learn (https://scikit-learn.org/) and Matplotlib (https://matplotlib.org/) packages of Python were used to perform LASSO regression model analysis, as well as to plot ROC curves, Rad-score map, and calibration curves. The multivariate binary logistic regression and nomogram construction were performed in R using the rms (Regression Modeling Strategies) package. The generalhoslem and rmda packages of R were used to perform Hosmer-Lemeshow test and plot decision curves, respectively.

## Results

### Patient Characteristics

In this study, a total of 255 patients with ccRCC from two hospitals was enrolled. The AHZMU included 132 ccRCC patients (81 male, 51 female; median age, 56 years [range, 11–85 years) as the training cohort, including 51 cases of tumor necrosis (38.6%). The GZPPH included 123 ccRCC patients (76 male, 47 female; median age, 56 years [range 23–80 years]) as the independent validation cohort, including 37 cases of tumor necrosis (30.0%). Demographic, pathological characteristics, and subjective image features of all patients are summarized in Table 1. Except for imaging necrosis (*P* < 0.001) and intra-tumoral vessels (*P* < 0.035), there were no statistically significant differences in other clinical and image information (*P* = 0.060–0.870).

**Table 1:** Characteristics of CCRCC Patients in the Training and Validation Cohorts.

**Characteristics**	**Training cohort (*****n*** **=** **132)**			**Validation cohort (*****n*** **=** **123)**			***P*-value**
	**Tumor necrosis (*n* = 51)**	**Non-tumor necrosis (*n* = 81)**	***P*-value**	**Tumor necrosis (*n* = 37)**	**Non-tumor necrosis (*n* = 86)**	***P*-value**	
Age, mean SD	56.02 ± 15.78	56.99 ± 11.60	0.861	56.30 ± 11.78	55.91 ± 13.27	0.877	0.632
Gender (%)			0.067			0.69	1
Male	26 (50.98%)	55 (67.90%)		24 (64.86%)	52 (60.47%)		
Female	25 (49.02%)	26 (32.10%)		13 (35.14%)	34 (39.53%)		
Tumor size, mean SD	6.39 ± 1.82	4.34 ± 2.02	<0.001^*^	6.30 ± 2.23	4.66 ± 2.10	<0.001^*^	0.924
Tumor boundary (%)			0.046^*^			0.002^*^	0.408
Circumscribed	39 (76.47%)	73 (90.12%)		23 (62.16%)	76 (88.37%)		
Infiltrative	12 (23.53%)	8 (9.88%)		14 (37.84%)	10 (11.63%)		
Necrosis imaging (%)			1			0.425	<0.001^*^
Absent	20 (39.22%)	33 (40.74%)		4 (10.81%)	15 (17.44%)		
Present	31 (60.78%)	48 (59.26%)		33 (89.19%)	71 (82.56%)		
Renal vein invasion (%)			0.285			0.007^*^	0.554
Absent	42 (82.35%)	73 (90.12%)		29 (78.38%)	82 (95.35%)		
Present	9 (17.65%)	8 (9.88%)		8 (21.62%)	4 (4.65%)		
Collecting system invasion (%)			0.001^*^			<0.001^*^	1
Absent	32 (62.75%)	71 (87.65%)		21 (56.76%)	75 (87.21%)		
Present	19 (37.25%)	10 (12.35%)		16 (43.24%)	11 (12.79%)		
Intratumoral vessels (%)			<0.001^*^			0.091	0.035^*^
Absent	3 (5.88%)	41 (50.62%)		4 (10.81%)	22 (25.58%)		
Present	48 (94.12%)	40 (49.38%)		33 (89.19%)	64 (74.42%)		
lymphatic metastasis (%)			0.045^*^			0.009^*^	0.153
Absent	44 (86.27%)	78 (96.30%)		27 (72.97%)	79 (91.86%)		
Present	7 (13.73%)	3 (3.70%)		10 (27.03%)	7 (8.14%)		
Visual relative enhancement (%)			0.509			0.835	0.171
Hyperattenuating	6 (11.76%)	8 (9.88%)		7 (18.92%)	15 (17.44%)		
Isoattenuating	31 (60.78%)	57 (70.37%)		22 (59.46%)	48 (55.81%)		
Hypoattenuating	14 (27.45%)	16 (19.75%)		8 (21.62%)	23 (26.74%)		
Enhancement pattern (%)			0.434			0.063	0.406
Homogeneous enhancement	14 (27.45%)	31 (38.27%)		7 (18.92%)	31 (36.05%)		
Relatively homogeneous enhancement	17 (33.33%)	24 (29.63%)		14 (37.84%)	34 (39.53%)		
Heterogeneous enhancement	20 (39.22%)	26 (32.10%)		16 (43.24%)	21 (24.42%)		
WHO/ISUP grading (%)			0.035^*^			<0.001^*^	0.667
I	4 (7.84%)	19 (23.46%)		0 (0.00%)	16 (18.60%)		
II	29 (56.86%)	48 (59.26%)		15 (40.54%)	64 (74.42%)		
III	15 (29.41%)	12 (14.81%)		17 (45.95%)	5 (5.81%)		
IV	3 (5.88%)	2 (2.47%)		5 (13.51%)	1 (1.16%)		
T stage (%)			<0.001^*^			<0.001^*^	0.709
T1	28 (54.90%)	71 (87.65%)		16 (43.24%)	72 (83.72%)		
T2	19 (37.25%)	5 (6.17%)		14 (37.84%)	10 (11.63%)		
T3	4 (7.84%)	5 (6.17%)		6 (16.22%)	4 (4.65%)		
T4	0 (0.00%)	0 (0.00%)		1 (2.70%)	0 (0.00%)		
N stage (%)			1			0.34	0.093
N0	4 (7.84%)	6 (7.41%)		4 (10.81%)	13 (15.12%)		
N1	2 (3.92%)	3 (3.70%)		1 (2.70%)	0 (0.00%)		
Nx	45 (88.24%)	72 (88.89%)		32 (86.49%)	73 (84.88%)		
M stage (%)			0.335			0.009^*^	0.321
M0	45 (88.24%)	76 (93.83%)		32 (86.49%)	85 (98.84%)		
M1	6 (11.76%)	5 (6.17%)		5 (13.51%)	1 (1.16%)		
TNM stage (%)			<0.001^*^			<0.001^*^	0.813
I	24 (47.06%)	66 (81.48%)		16 (43.24%)	72 (83.72%)		
II	17 (33.33%)	5 (6.17%)		12 (32.43%)	9 (10.47%)		
III	4 (7.84%)	6 (7.41%)		4 (10.81%)	4 (4.65%)		
IV	6 (11.76%)	4 (4.94%)		5 (13.51%)	1 (1.16%)		

* *P < 0.05 means statistical significance*.

*Data are in n (%) unless otherwise indicated*.

*Categorical variables are compared using chi-square tests or Fisher exact tests, while continuous variables are compared using t-test or Mann–Whitney U-test, as appropriate*.

### Image Features Model Construction

Univariate analysis of demographic and subjective image features in both cohorts are summarized in Table 1. Univariate analysis revealed that tumor size, tumor margin, intra-tumoral vessels, invasive system infiltration, lymph node metastasis, and tumor necrosis were closely related (*P* < 0.05) in the training cohort. However, after multivariate analysis, only tumor size (OR 1.404 [95% CI 1.129–1.795]; *P* < 0.001) and intratumoral vessels (OR 8.044 [95% CI 2.407–36.971]; *P* = 0.002) remained as independent predictors. Therefore, the image features model was constructed by integrating tumor size and intratumoral vessels, which yielded an AUC of 0.82 (95% CI 0.75–0.89) in the training cohort and 0.72 (95% CI 0.62–0.82) in the validation cohort.

### Radiomics Signature Construction

A total of 2,436 radiomics features were extracted from the corticomedullary and nephrographic phase contrast-enhanced CT images, and used for feature selection simultaneously. After removing redundant features by consistency analysis, 1,194 radiomics features remained for the corticomedullary phase and 1,189 for the nephrographic phase (ICC > 0.75). Subsequently, 37 robust radiological features with non-zero coefficients (26 corticomedullary and 11 nephrographic features) were screened using the LASSO logistic regression model. Finally, a Rad score formula was constructed based on the above features and their corresponding weighting coefficients (i.e., radiomics signature), as shown in Section S2. A Rad score could be calculated for each patient in the training and validation cohorts using this formula, with no significant difference in Rad score between the two cohorts (*P* = 0.648), while with significant differences between the tumor necrosis group and non-tumor necrosis group in both cohorts, as shown in Table 2. The optimal cut-off value, based on the Youden Index Rad score, was 0.313. Radiomics signatures demonstrated AUCs of 0.91 (95% CI 0.87–0.96) and 0.86 (95% CI 0.79–0.93) in the training and validation cohorts, respectively.

**Table 2:** Rad-score in the Training and Validation Cohorts.

	**Training cohort (*****n*** **=** **132)**		***P*-value**	**Validation cohort (n=123)**		***P*-value**	***P*-value**
	**Tumor necrosis (*n* = 51)**	**Non-tumor necrosis (*n* = 81)**		**Tumor necrosis (*n* = 37)**	**Non-tumor necrosis (*n* = 86)**		
Rad-score	0.577 (0.187 to 1.193)	0.224 (-0.006 to 0.732)	<0.001	0.533 (0.057 to 1.100)	0.201 (-0.054 to 0.717)	<0.001	0.6478

### Radiomics Nomogram Construction

**Figure 3:**
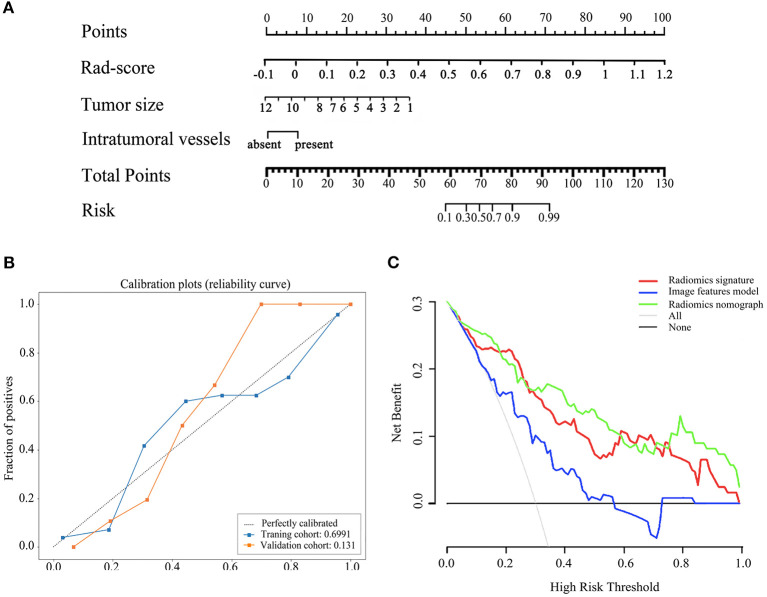
The radiomics nomogram, calibration curves of the radiomics nomogram and decision curve analysis. The radiomics nomogram was established based on radiomics signature, tumor size, intratumoral vessels in the training cohort **(A)**. Calibration curves of the radiomics nomogram in the training and validation cohorts **(B)**. The y-axis expresses the actual tumor necrosis rate, the x-axis expresses the predicted possibility and the 45°gray dotted line expresses the ideal prediction. Calibration curves demonstrated the goodness-of-fit of the radiomics nomogram. Decision curve analysis for three model. Decision curve analysis DCA) for each model in the validation dataset **(C)**. The DCA demonstrated that if the threshold probability was >5%, the application of radiomics nomogram to predict tumor necrosis adds more benefit than treating all or none of the patients, radiomics signatrue and image features model.

By integrating radiomics signature (OR 4.472 [95% CI 0.289–8.654]; *P* = 0.048), tumor size (OR 0.550 [95% CI−1.981–3.080]; *P* = 0.019), and intra-tumoral vessels (OR 4.472 [95% CI 0.289–8.654]; *P* = 0.048), a radiomics nomogram was built in the training cohort, as shown in Figure 3A. The VIF of radiomics signature, tumor size, and intra-tumoral vessels are 2.177, 2.202, and 1.458 in radiomic nomogram respectively, which demonstrated there was a multicollinearity between radiomic signature and tumor size, but not serious. In the nomogram, the radiomics signature demonstrated the highest weight, indicating it was the most important predictive factor for tumor necrosis. The radiomics nomograph demonstrated satisfactory predictive performance, with AUCs of 0.93 (95% CI 0.89–0.97) and 0.87 (95% CI 0.81–0.94) in the training and validation cohorts, respectively. The calibration curve revealed that the radiomics nomogram demonstrated good agreement between the predicted probability and the expected probability, and the Hosmer–Lemeshow test demonstrated good similarity in the training (*p* = 0.695) and validation (*p* = 0.131) cohorts, as shown in Figure 3B.

### Model Evaluation

**Figure 4:**
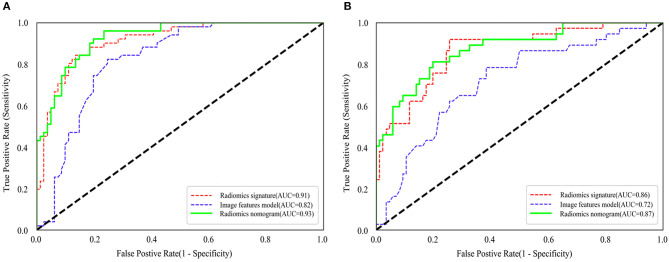
Comparison of ROC curves between radiomics nomogram, image features model and radiomics nomogram for prediction of tumor necrosis in the training cohort **(A)** and validation cohort **(B)**. The three colors of the curves represent different models: red, radiomics signature; blue, image features model; green, radiomics nomogram.

The ROC curves of the three models for prediction of tumor necrosis are shown in Figure 4, while predictive performance (AUC, sensitivity, specificity, and accuracy) is summarized in Table 3. The predictive performance of the radiomics signature was superior to the image features in both cohorts. After combining radiomics signature with tumor size and intra-tumoral vessels to construct the radiomics nomogram, the predictive performance of the clinical model was significantly improved in the training cohort (from 0.82 to 0.93; *P* < 0.001). This significant improvement was also verified in the validation cohort (from 0.72 to 0.87; *P* = 0.001), indicating that the radiomics signature had a gain value for the prediction of the image features model. The AUC of the radiomics nomogram was also slightly higher than the radiomics signature, although the difference was not statistically significant (*P* = 0.222 [training cohort], *p* = 0.425 [validation cohort]).

**Table 3:** Predictive performance of the image features model, the radiomics signature, and the radiomics nomogram.

**Model**	**Training cohort (*****n*** **=** **132)**				**Validation cohort (*****n*** **=** **123)**			
	**AUC (95% CI)**	**Sensitivity**	**Specificity**	**Precision**	**AUC (95% CI)**	**Sensitivity**	**Specificity**	**Precision**
Image features model	0.82 (0.75–0.89)	86.42%	84.31%	85.61%	0.72 (0.62–0.82)	59.30%	78.38%	65.04%
Radiomics Signature	0.91 (0.87–0.96)	75.31%	82.35%	78.03%	0.86 (0.79–0.93)	82.56%	70.27%	78.86%
radiomics nomogram	0.93 (0.89–0.97)	76.54%	96.08%	84.09%	0.87 (0.81–0.94)	72.26%	83.78%	76.42%

The clinical decision curve is presented in Figure 3C, which shows that when the threshold probability was >5%, the radiomics signature was higher or similar to the radiomics nomogram in the partial threshold probability. However, within most of the above threshold probabilities, the radiomics nomogram demonstrated a larger net benefit than did the radiomics signature, indicating that the nomogram had the best clinical utility for prediction of tumor necrosis in patients with ccRCC.

## Discussion

To our knowledge, this was the first study to develop and validate a CT-based radiomics signature to preoperatively predict tumor necrosis in patients with ccRCC from two different centers. The results demonstrated that the predictive performance of the radiomics signature was significantly superior to the image features model. In addition, by integrating the radiomics signature and significant imaging features, an easy-to-use radiomics nomogram was established to facilitate individualized preoperative prediction with the best performance, which is expected to provide valuable information to support clinical decision making.

An image features model based on tumor size and intratumoral vessels was developed first, which demonstrated good predictive performance for tumor necrosis in ccRCC in both cohorts. Tumor size in the model, as a primary predictor, suggested that larger tumors are more prone to tumor necrosis, which is consistent with previous studies in which tumor size was reported to be a significant independent factor for invasive biological behavior of ccRCC (5). In addition, the results show that the imaging findings of intratumoral vessels are more common in ccRCC with tumor necrosis, which may be related to the mechanism of tumor necrosis; more specifically, excessive blood supply, immature blood vessels, and hypoxia associated with vascular remodeling in the tumor (7, 22, 23). Therefore, intratumoral vessels can be used to indicate the presence of tumor necrosis in patients with ccRCC.

Tumor necrosis is a major cause of image heterogeneity in patients with ccRCC. It is challenging to visually assess this heterogeneity because there are significant differences in the size, morphology, and degree of enhancement of tumor necrotic areas in CT images (24). However, the emergence of radiomics provides a new approach to solve this problem, which hypothesizes that medical images contain numerous and important phenotypic information invisible to the naked eye, and the relationship between imaging data and tumor characteristics can be uncovered through deep mining and quantitative analysis of imaging data (14). As reported by Aerts et al., intratumor heterogeneity can be described by radiomics (25). This hypothesis was also proven by the results of this study, in which the radiomics signature outperformed the image features model in predicting tumor necrosis in ccRCC. Consistent with previous studies, the radiomics signature consists mainly of three-dimensional texture features, and its prediction performance was significantly better than that of morphological features and first-order features (26, 27). The reason is that the three-dimensional texture features can provide gross characterization of tumor heterogeneity through analysis of the distribution and relationship with gray levels of pixels or voxels in CT images (28). In addition, radiomics features are better than image features with regard to repeatability and robustness by automating high-throughput feature extraction algorithms, thus avoiding intra- and interobserver disagreement. Both the training and validation cohorts demonstrated good predictive consistency, indicating that radiomics signatures have better generalization ability between different centers. In summary, objective and quantitative radiomics analysis offers a new approach to the assessment of tumor invasiveness in ccRCC.

To explore clinical use, further incorporating the radiomics signature, tumor size and intratumoral vessels, a radiomics nomograph was established to preoperatively evaluate the tumor necrosis risk for each ccRCC patient, which achieved significantly and slightly improved performance compared with imaging features and radiomics models alone. Unexpectedly, the tumor size were negatively correlated with tumor necrosis in the radiomic nomogram, which opposited of that in image feature model. Radiomic signature contains a radiomic feature representing the maximum diameter of the tumor on the coronal plane, that is, the Maximum_2D_Diameter_Row. Based on the VIF, there was some multicollinearity between radiomic signature and tumor size, but not serious. Therefore, we think that the tumor size may be weakened in the risk forecast weight and shows an opposite prediction trend for tumor necrosis, but it still play an important role in the model optimization. Moreover, decision curve analysis demonstrated that more net benefits within the most of thresholds probabilities could be achieved using the radiomics nomograph, meaning that using the nomogram for therapy strategy would achieve a better clinical outcome. Therefore, a radiomics nomograph can be regarded as a promising assistive tool to guide clinical management in ccRCC patients for radiologists and oncologists.

Biopsy is the primary method for preoperative diagnosis of tumor necrosis in patients with ccRCC; however, it is limited by its invasiveness and potential for complications. In addition, the accuracy of diagnosing tumor necrosis through biopsy is poor due to tumor heterogeneity and sampling error (11). In contrast, a radiomics nomogram demonstrates better performance in the preoperative discrimination of tumor necrosis, given the advantage in characterization of spatial heterogeneity of the entire tumor. In addition, a radiomics nomogram with quantitative analysis and non-invasive examination can be used as a simple, well-accepted method for longitudinal assessment of tumor progression. Therefore, although radiomics is currently not an alternative to biopsy for the assessment of tumor necrosis, it can provide an important reference or Supplementary Materials.

There were several notable limitations to our study. Although this study used an independent patient population as a validation cohort, the radiomics nomogram should be further validated in a prospective study with a larger dataset. Due to the two-center nature of the study, differences in the diagnosis of tumor necrosis and the CT scan protocols were unavoidable, which may have led to inherent bias. Different proportions of necrosis have different prognostic value (29); however, this study only explored the performance of radiomics signature in discrimination of tumor necrosis. ROI segmentation is an important preprocessing step in radiomics analysis; as such, automated or semi-automated segmentation is expected to improve the robustness of the radiomics model.

In conclusion, this study proposed a radiomics nomogram for preoperative assessment of tumor necrosis in patients with ccRCC, which demonstrated satisfactory performance. As a non-invasive, efficient, quantitative approach, a radiomics signature can add incremental value to imaging features for assessment of tumor invasiveness and facilitate preoperative clinical decision making and/or management of patients with ccRCC.

## Data Availability Statement

The experimental datasets in this study are not publicly available due to patient privacy concerns but can be obtained from the corresponding author with reasonable request, pending ethical approval by the institutional review board of Guizhou Provincial People's Hospital.

## Ethics Statement

This study was approved by the Ethics Review Committee of Guizhou Province People's Hospital. Requirements for written informed consent were waived by the committee due to the retrospective nature of the study.

## Author Contributions

Guarantor of integrity of the entire study: RW and TZ. Study concepts and design: RW, TZ, and CL. Literature research: YJ. Assessment of Image feature: CT, XZ, HL, and WL. Assessment of pathological indicators: YCa, YCh, YY, and YB. Statistical analysis: YJ, CH, and YL. Analysis and interpretation of data: YJ, QC, and WL. Drafting the article: YJ. Manuscript editing: YJ and RW.

## Conflict of Interest

CH and YL were employed by the Beijing Deepwise & League of PHD Technology Co. Ltd. The remaining authors declare that the research was conducted in the absence of any commercial or financial relationships that could be construed as a potential conflict of interest.

## Abbreviations

CC: Renal cell carcinoma
ccRCC: Clear cell renal cell carcinoma
SSIGN, Stage, Size: Grade and Necrosis
CT: Computed tomography
GZPPH: Guizhou Province People's Hospital
AHZMU: Affiliated hospital of Zunyi Medical University
WHO: World Health Organization
ISUP: International Urological Pathology Association
ROI: Region of interest
LASSO: Least absolute shrinkage and selection operator
ICC: Intraclass correlation coefficient
VIF: Variance Inflation Factor.
