# Supplementary material for: A Computed Tomography-Based Radiomics Nomogram to Preoperatively Predict Tumor Necrosis in Patients With Clear Cell Renal Cell Carcinoma
Source: Front Oncol. 2020 May 29;10:592. doi: 10.3389/fonc.2020.00592 (PMC7272670; doi:10.3389/fonc.2020.00592)
Supplement: Supplementary file 1 [file Data_Sheet_1.DOC]

# Supplementary Materials

## Section 1 CT equipment and parameters

Siemens' first generation dual source CT (Definition Flash, Siemens Healthcare, Forchheim, Germany) and third generation dual source CT (Force, Siemens Healthcare, Forceheim, Germany) were adopted in Guizhou Province People’s Hospital. Both the two CTs introduced the optimal automatic adjustment technology of CARE kV tube voltage and CARE Dose4D tube current, with reference tube voltages being 120 kV and 100 kV, respectively, and the reference tube currents being 300 mAs and 200 mAs respectively. And the collimator widths of the two CTs were 64×0.6 mm and 192×0.6 mm, respectively. The remaining scanning parameters were the same, specifically including FOV: 350 mm×350 mm (FOV value varies with body size, ranging from 300mm to 350mm); matrix: 512 × 512; screw pitch: 1; rotation time: 0.5 seconds; and reconstruction layer thickness: 5 mm. The contrast agent (Ultravat 370, Bayer Schering, Berlin, Germany, 2.5-3.5 mL/s, 1-1.5 mL/kg, the total amount of contrast agent and the injection rate are selected according to the patient's specific conditions) was injected through the cubital vein by a high pressure syringe pump, followed by the injection of 20 ml of normal saline at the same flow rate. After the contrast agent was injected, the renal corticomedullary phase (25 ~ 30s), nephrographic phase (60 ~ 90s), excretory phase (5 ~ 7min) were scanned.

The Affiliated hospital of Zunyi Medical University adopted Siemens' twice generation dual source CT (Somatom Definition Flash, Siemens Healthcare, Forchheim, Germany) and GE Optima CT680 (GE Medical, USA). The Siemens CT scan introduced introduced the optimal automatic adjustment technology of CARE kV tube voltage and CARE Dose4D tube current with reference tube voltages being 100 kV and reference tube current is 350 mAs. The GE CT Tube current modulation technique with a reference tube voltage of 120 kV and a reference tube current of 180 mAs. the pitch is 0.5 and 1.375, and the rotation time is 0.5 s and 0.8 s in the Siemens and GE CT scan, respectively. The remaining scanning parameters are the same: FOV 350 mm × 350 mm (FOV value varies with body type, range is 300mm-350mm); matrix 512 × 512; collimator width 128 × 0.6 mm; reconstruction layer thickness 5 mm. The contrast agent (3.5 mL / s, 1.5 mL / kg, Ultravat 370, Bayer Schering, Berlin, Germany) was injected through the cubital vein by a high pressure syringe pump, followed by the injection of 20 ml of normal saline at the same flow rate. After the contrast agent was injected, the scanning over renal cortex (25 ~ 30s), renal parenchyma (60 ~ 90s), renal pelvic excretion (5 ~ 7min) were performed.

## Section 2 Radiomics score formula

Rad-score = 0.345098+

original_firstorder_Variance_CMP * -0.043235 +

wavelet-LHL_glrlm_LongRunLowGrayLevelEmphasis_NP * -0.039479 +

log-sigma-1-0-mm-3D_glszm_SmallAreaLowGrayLevelEmphasis_CMP * -0.034671 +

wavelet-LHH_glcm_ClusterShade_NP * -0.026584 +

log-sigma-5-0-mm-3D_firstorder_Maximum_CMP * -0.018278 +

wavelet-HHH_gldm_SmallDependenceLowGrayLevelEmphasis_CMP * -0.014365 +

wavelet-LLL_glszm_LargeAreaLowGrayLevelEmphasis_CMP * -0.009158 +

original_firstorder_Median_CMP * -0.008041 +

wavelet-HHH_glszm_GrayLevelNonUniformity_CMP * -0.006089 +

log-sigma-5-0-mm-3D_gldm_SmallDependenceEmphasis_CMP * -0.005242 +

log-sigma-4-0-mm-3D_glcm_Imc1_CMP * -0.002172 +

original_gldm_GrayLevelVariance_CMP * -0.002033 +

wavelet-HHL_firstorder_Median_CMP * 0.000125 +

wavelet-HLL_glcm_ClusterShade_CMP * 0.000971 +

wavelet-HHH_glszm_SizeZoneNonUniformityNormalized_NP * 0.001879 +

wavelet-HHL_glcm_Idn_CMP * 0.001955 +

log-sigma-4-0-mm-3D_firstorder_Median_CMP * 0.003731 +

log-sigma-4-0-mm-3D_glszm_SmallAreaLowGrayLevelEmphasis_CMP * 0.004768 +

wavelet-LHH_firstorder_Median_CMP * 0.005871 +

original_gldm_LargeDependenceLowGrayLevelEmphasis_NP * 0.006435 +

log-sigma-4-0-mm-3D_glcm_MaximumProbability_NP * 0.006945 +

log-sigma-5-0-mm-3D_glszm_GrayLevelNonUniformity_NP * 0.007121 +

wavelet-HLL_firstorder_Skewness_NP * 0.007438 +

original_shape_Maximum2DDiameterRow_NP * 0.007756 +

log-sigma-5-0-mm-3D_gldm_DependenceVariance_NP * 0.009785 +

log-sigma-5-0-mm-3D_glrlm_RunLengthNonUniformity_NP * 0.013696 +

wavelet-LHH_glszm_SizeZoneNonUniformityNormalized_NP * 0.015217 +

log-sigma-5-0-mm-3D_firstorder_90Percentile_CMP * 0.01524 +

wavelet-HLH_glszm_SmallAreaEmphasis_NP * 0.015304 +

log-sigma-1-0-mm-3D_firstorder_Skewness_NP * 0.019005 +

wavelet-LHL_glcm_Idmn_NP * 0.019507 +

wavelet-LLH_glszm_SizeZoneNonUniformityNormalized_NP * 0.021552 +

wavelet-LLH_glszm_SmallAreaEmphasis_CMP * 0.021625 +

wavelet-HHH_glszm_SizeZoneNonUniformityNormalized_CMP * 0.03011 +

wavelet-LLH_glszm_LargeAreaLowGrayLevelEmphasis_NP * 0.034384 +

log-sigma-5-0-mm-3D_glrlm_RunLengthNonUniformity_CMP * 0.04684 +

log-sigma-4-0-mm-3D_glszm_GrayLevelNonUniformity_CMP * 0.086433
